# Biomonitoring of Multiple Mycotoxins in Urine by GC–MS/MS: A Pilot Study on Patients with Esophageal Cancer in Golestan Province, Northeastern Iran

**DOI:** 10.3390/toxins13040243

**Published:** 2021-03-29

**Authors:** Farhad Niknejad, Laura Escrivá, Khoda Berdi Adel Rad, Masoud Khoshnia, Francisco J. Barba, Houda Berrada

**Affiliations:** 1 Laboratory Sciences Research Center, Golestan University of Medical Sciences, Gorgan 4918936316, Iran; niknejad@goums.ac.ir; 2 Department of Preventive Medicine and Public Health, Food Science, Toxicology and Forensic Medicine, Faculty of Pharmacy, University of Valencia, 46100 Burjassot, València, Spain; laura.escriva@uv.es; 3 Student Research Committee, Golestan University of Medical Sciences, Gorgan 4918936316, Iran; adeljamshid9@gmail.com; 4 Digestive Oncology Research Center, Digestive Disease Research Institute, Tehran University of Medical Sciences, 14535 Tehran, Iran; khoshniamd@gmail.com

**Keywords:** mycotoxins, esophageal cancer, urine biomarkers, GC–MS/MS

## Abstract

A pilot study to investigate the occurrence of 10 mycotoxins (deoxynivalenol, DON; 3-acetyldeoxynivalenol, 3-ADON; 15-acetyldeoxynivalenol, 15-ADON; fusarenon-X, FUS-X; diacetoxyscirpenol, DAS; nivalenol, NIV; neosolaniol, NEO; zearalenone, ZON; zearalanone, ZAN; T-2 toxin, T-2; and HT-2 toxin, HT-2) in esophageal cancer patients was performed with the urinary biomarkers approach in Golestan, Iran. Urine multimycotoxin analysis was performed by dispersive liquid–liquid microextraction and gas chromatography–tandem mass spectrometry (GC–MS/MS) analysis, and values were normalized with urinary creatinine (μg/g). Four mycotoxins, namely NEO (40%), HT-2 (17.6%), DON (10%), and HT-2 (5.8%), were detected in the analyzed urine samples. DON was only detected in the control group (5.09 μg/g creatinine), while T-2 (44.70 μg/g creatinine) was only present in the esophageal cancer group. NEO and HT-2 were quantified in both control and case groups, showing average of positive samples of 9.09 and 10.45 μg/g creatinine for NEO and 16.81 and 29.09 μg/g creatinine for HT-2, respectively. Mycotoxin co-occurrence was observed in three samples as binary (NEO/HT-2 and T-2/HT-2) and ternary (DON/NEO/HT-2) combinations, reaching total concentrations of 44.58, 79.13, and 30.04 µg/g creatinine, respectively. Further investigations are needed to explore a causal association between mycotoxin contamination and esophageal cancer. For this pilot study in Golestan, the low sample size was a very limiting factor.

## 1. Introduction

Mycotoxins are toxic secondary metabolites naturally produced by fungal species that commonly contaminate food and feed [1]. Human exposure to mycotoxins is largely through the consumption of contaminated food; however, other mycotoxin sources such as occupational exposure and inhalation need to be considered [2]. Mycotoxins are harmful to human health as they may cause several toxic effects, which include teratogenicity, nephrotoxicity, hepatotoxicity, immunotoxicity, hematotoxicity, and hormonal alterations and reproductive effects [3,4]. Some mycotoxins have been classified as carcinogenic to humans (aflatoxins B1, B2, G1, and G2) or possibly carcinogenic to humans (ochratoxin A, aflatoxin M1, fumonisins B1 and B1) by the International Agency for Research on Cancer (IARC) [5]. Deoxynivalenol (DON) also causes acute effects such as nausea, diarrhea, reduced nutritional efficiency, gastrointestinal tract injuries, and weight loss in animals [6]. Several countries have implemented regulations in food products and raw materials aiming to limit mycotoxin exposure and to protect consumers’ health [7].

In recent years, human biomonitoring has been recognized as an efficient and cost-effective approach to assessing humans’ exposure to mycotoxins [8]. Biomarkers are considered a useful tool to evaluate mycotoxin exposure at the individual level while avoiding dietary registration as well as the limitations associated with the heterogeneous contamination of mycotoxins in food, which otherwise may hinder appropriate exposure assessments [9,10]. Knowledge of mycotoxins’ effects on the human metabolism is fundamental to performing exposure assessment studies using the biomarkers approach. Typical biomarkers of exposure include the toxins themselves and/or their main products from phase I and phase II metabolization (e.g., conjugation with glucuronic acid). Those compounds are generally determined in biological fluids such as plasma, serum, or urine [11], the last being commonly preferred for mycotoxin biomonitoring and screening purposes since it is easy to collect by noninvasive procedures and large amounts of sample can be obtained. Moreover, according to toxicokinetic studies in animals, most of the mycotoxins are excreted through the kidneys within 48 h. Therefore, the levels of urinary biomarkers are useful to provide updated information about the recent intake of these toxic compounds [12]. However, a major limitation is the extremely low analyte concentrations detected in urine after dietary exposure; therefore, analytical methods must be sensitive enough to detect low mycotoxin levels, as well as their metabolites [13]. Furthermore, considering the chronic dietary exposure to potentially contaminated foods, as well as the high variation in the human diet, people are commonly exposed to several mycotoxins at the same time, so reproducing situations of real exposure requires simultaneous assessment of different mycotoxins [11].

Golestan Province, located in northeastern Iran, has been recognized as a high-risk area for esophageal cancer [14], a devastating disease with a poor prognosis [15]. Different risk factors have been associated with esophageal cancer in this region, including opium consumption, hot tea intake, limited oral hygiene, obesity, exposure to polycyclic aromatic hydrocarbons, and genetic factors [16]. Epidemiological studies suggested a relationship between intake of mycotoxin-contaminated food and esophageal cancer. Studies in South Africa and China reported that communities that consume the most maize and/or wheat in those countries have elevated rates of esophageal cancer [17,18,19]. The high prevalence of esophageal cancer in Western Kenya and its associated factors were investigated, and the most plausible cause was reported to be mycotoxins, particularly fumonisins present in food as a result of poor manufacturing of cereals, especially maize [20]. A direct association between maize consumption and esophageal cancer was shown in Northeast Italy [21]. According to studies in Iran, South Africa, and China, higher exposures to fumonisins were observed in regions where the risk of esophageal cancer was higher [22]. In Golestan, high levels of fumonisin B1 were found in corn and rice samples with a significant positive relationship between rice contamination with mycotoxins and esophageal cancer risk [23]. Moreover, a positive relationship between aflatoxin levels in wheat flour samples and the risk of esophageal cancer in Iran was reported [24]. However, there are no data concerning the possible association between urinary mycotoxin levels and the incidence of esophageal cancer in this high-risk area.

The aim of this study was to determine 10 mycotoxins, namely DON, 3-acetyldeoxynivalenol (3-ADON), 15-acetyldeoxynivalenol (15-ADON), fusarenon-X (FUS-X), nivalenol (NIV), neosolaniol (NEO), zearalenone (ZON), zearalanone (ZAN), T-2 toxin (T-2), and HT-2 toxin (HT-2), in urine samples from esophageal cancer patients as a case group and their healthy close relatives as a control group. This survey serves as the first study to examine multimycotoxin urinary levels in both healthy volunteers and esophageal cancer patients from Golestan Province of northeastern Iran by gas chromatography–tandem mass spectrometry (GC–MS/MS).

## 2. Results and Discussion

### Sample Analyses

Of the 10 investigated mycotoxins, four were detected and quantified in the analyzed urine samples, namely DON, NEO, T-2, and HT-2 toxins, while NIV, 3-ADON, 15-ADON, FUS-X, ZON, and ZAN were not detected in any sample. Four control samples (4/10) were positive for mycotoxins. DON and HT-2 were detected in one control sample each (1/10), showing values of 8.42 ± 1.21 µg/L and 23.97 ± 9.97 µg/L, respectively, while four (4/10) control samples were positive for NEO with a mean value of 14.15 ± 5.72 µg/L and reached a maximum concentration of 22.53 ± 2.39 µg/L. The creatinine-corrected mean values for DON, HT-2, and NEO for positive samples in the control group were 5.90, 16.81, and 10.45 µg/g creatinine, respectively.

Regarding the esophageal cancer group, three samples (3/17) were positive for three of the analyzed mycotoxins (NEO, T-2, and HT-2), while DON was not detected in any urine samples from the esophageal cancer patients. NEO and T-2 were detected in one sample each (1/17), with values of 12.90 ± 3.87 µg/L (9.09 µg/g creatinine) and 50.09 ± 7.51 µg/L (44.70 µg/g creatinine), respectively. HT-2 was detected in the three positive samples (3/17) with a mean value of 36.52 ± 16.07 µg/L (29.09 µg/g creatinine) and reached a maximum concentration of 50.38 ± 22.54 µg/L. Table 1 shows mycotoxin incidence; minimum, maximum, and mean mycotoxin concentrations (µg/L) with SD; and creatinine-corrected mean values (µg/g creatinine) of each detected mycotoxin in positive samples of both the control group and the esophageal cancer case group.

**Figure 1:**
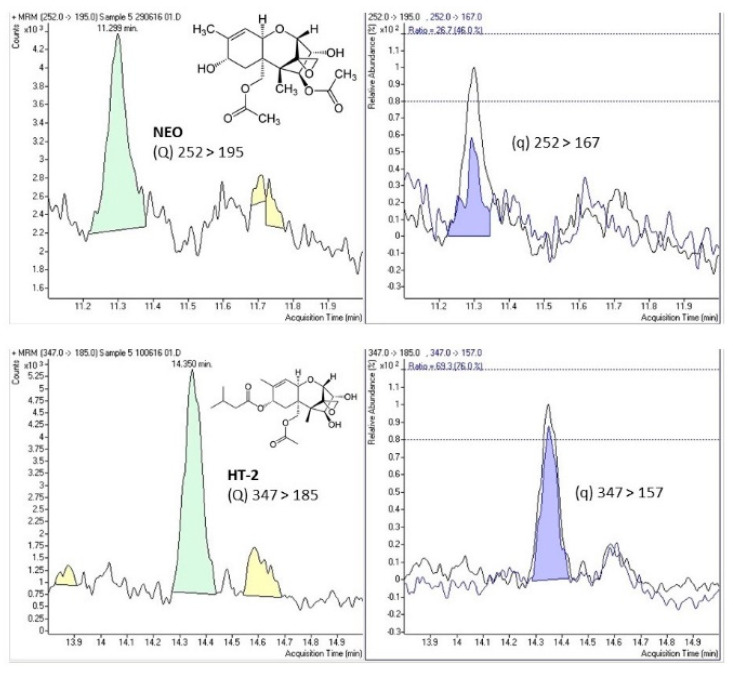
Selected-reaction monitoring (SRM) chromatograms of a case study sample positive for neosolaniol (NEO) and HT-2 toxin (HT-2) indicating the chemical structure and the quantitation (Q) and confirmation (q) transitions for both compounds.

Figure 1 shows the selected-reaction monitoring (SRM) chromatograms of a case study sample positive for NEO and HT-2, indicating the chemical structure and both transitions (quantitation (Q) and confirmation (q)) for the detected mycotoxins.

Overall, DON was only detected in the control group, while T-2 toxin was only present in the esophageal cancer group. However, NEO and HT-2 were quantified in both control and esophageal cancer groups, being the most detected mycotoxins in the control (40%) and the esophageal cancer group (5.8%), respectively. When comparing the positive samples of control and esophageal cancer groups, HT-2 mean urine values, as well as creatinine-normalized levels, were higher in the esophageal cancer group (36.52 ± 16.07 µg/L; 29.09 µg/g creatinine) than in the control group (23.97 ± 9.97 µg/L; 16.81 µg/g creatinine). NEO instead showed slightly lower urine mean concentration in the case group (12.90 ± 3.87 µg/L; 9.09 µg/g creatinine) than the control group (14.15 ± 5.72 µg/L; 10.45 µg/g creatinine).

Among the positive samples, co-occurrence of more than one mycotoxin was found in three urine samples showing the binary combinations NEO/HT-2 and T-2/HT-2 in two samples from the case group and the ternary combination DON/NEO/HT-2 in one control sample. The sum of mycotoxin levels in co-contaminated samples reached values of 63.27 µg/L (44.58 µg/g creatinine) and 90.38 µg/L (79.13 µg/g creatinine) for NEO/HT-2 and T-2/HT-2, respectively, and the value of 42.82 µg/L (30.04 µg/g creatinine) for the ternary combination DON/NEO/HT-2. As shown in Table 2, HT-2 was the most common mycotoxin in combination present in all the co-occurrent samples, followed by NEO, which was present in two of the three multicontaminated urine samples.

Several pilot studies applying multibiomarker methods have shown regional differences in urinary biomarker excretion patterns in Europe, Asia, and Africa [25,26,27,28]. For instance, DON was highly common in urine samples from adult populations in European countries, including Sweden (>90%) [29], Norway (99%) [30], the United Kingdom (93–98.7%) [30,31], Italy (76–87%) [30,32], Spain (68.5–91%) [33,34], and Portugal (63%) [35]. DON was also reportedly the most prevalent urinary mycotoxin in Brazil (88%) [6] and South Africa (87%) [36].

Conversely, in the present study, DON was poorly detected (10%). The reasons for this variation are not clear, but differences in dietary patterns may be an important factor. The Iranian diet is very rich in wheat and rice, considered the main and most commonly consumed cereals; however, other cereals such as maize are less frequently consumed. DON incidence (10%) was also low in urine samples from China, where the most frequently detected mycotoxin (43.8%) was its metabolite deoxynivalenol-15-glucuronide (DON-15-GlcA) [37]. Nevertheless, urinary DON and DON-glucuronide were frequently detected (72%) in women from Golestan, northern Iran [38], indicating that other factors in dietary habits may influence DON urinary levels and mycotoxin excretion patterns. Comparing different studies is challenging since there are several variable factors such as differences in food habits, age, race, and sample size. DON was detected in 76% of urine samples in the Italian population, including children, adolescents, adults, the elderly, vegetarians, and pregnant women, revealing statistically significant differences in population groups, with the highest concentrations of total DON in children and adolescents and the lowest incidence (40–43%) in pregnant women [39]. On the other hand, conjugation with glucuronic acid was reported as the metabolization route of 80% of DON excreted in urine [39], and it has been suggested that de-epoxy-deoxynivalenol 1 (DOM-1) is one of the DON detoxifying routes in humans [40]; therefore, DON metabolites and its conjugation forms should be considered to estimate the total urinary DON by measuring both free and total (free + conjugated) DON before and after enzymatic treatment. The natural occurrence of DON and its metabolites was evaluated in human urine samples from Portugal; 15% of the samples were positive for free DON and 69% were positive for total DON. As in the present study, 3-ADON and 15-ADON were not detected in any of the analyzed samples [41].

The mycotoxins showing the highest incidence in the present work were NEO (40%), followed by HT-2 (17.6%) and T-2 (5.8%). The presence of T-2 and HT-2 was also reported in urine samples from France, Belgium, the Czech Republic, Norway, and the Netherlands (29.8% incidence), with a median of 93.5 ng/L and reaching urinary levels up to 39,600 ng/L; NEO showed low incidence (6.4%), reaching median and maximum values of 168 and 3390 ng/L, respectively [25]. Low incidence (2.3%) was reported for T-2 in urine samples from China [37], while HT-2 and T-2 toxins were not detected in urine samples from Brazil [6].

Exposure to mycotoxin combinations is of importance even at low doses since they may interact, resulting in toxic effects observed at lower concentrations than the individual mycotoxin effects, which may be difficult to predict [42]. In the present study, mycotoxin combination was not common, but it was observed in three samples, reaching considerable concentrations.

The present study identified slight differences between the esophageal cancer patients and the control group, showing for the first group higher HT-2 urine values compared to the control group. This could be indicative of an association between the mycotoxin and the disease; however, considering the small sample size, it was not possible to establish any causal relationship, and more studies in this area are needed. In the present study, the control group comprised healthy patients’ close relatives to minimize differences in dietary habits. It was assumed that each patient and their corresponding relatives had similar dietary patterns. Appreciable age difference among the control group (20–46, mean 33.5 years old) and case group (50–92, mean 69.1 years old) was registered. Differences in dietary patterns among different age groups could be a factor affecting mycotoxin occurrence. However, no significant differences through age stratification were observed. In this sense, similar results were reported in a large Aflatoxins biomonitoring study conducted in the United States on people ranging from 18 to 83 years old with a high incidence of hepatocellular carcinoma [43]. Moreover, since cancer is a multifactorial disease, the age factor could play a role in the development of the disease.

## 3. Conclusions

The determination of urinary biomarkers as a valuable approach to assessing the exposure to 10 mycotoxins in esophageal cancer patients and healthy close relatives was successfully carried out. The mycotoxins NEO (40%), HT-2 (17.6%), DON (10%), HT-2 (5.8%) were detected in the analyzed urine samples. DON was only detected in the control group, while T-2 toxin was only present in the esophageal cancer patients. NEO and HT-2 were quantified in both control and esophageal cancer patients, and differences were observed between both groups; HT-2 values were slightly higher in the esophageal cancer group (29.09 µg/g creatinine) compared to the control group (16.81 µg/g creatinine). Mycotoxin co-occurrence was observed in three samples as binary (NEO/HT-2 and T-2/HT-2) and ternary (DON/NEO/HT-2) combinations, reaching total concentrations of 44.58, 79.13, and 30.04 µg/g creatinine, respectively. Further studies with larger sample sizes are needed to examine the possible relationship between mycotoxin exposure and cancer incidence.

## 4. Material and Methods

### 4.1. Standards

Mycotoxin standards, including DON, 3-ADON, 15-ADON, FUS-X, DAS, NIV, NEO, ZAN, HT-2, and T-2, were purchased from Sigma-Aldrich (St. Louis, USA). Stock solutions (1000 mg/L, in methanol) of the individual mycotoxins were used to prepare the required multianalytes’ working standard solutions (50 mg/L, in acetonitrile). Standards were maintained in darkness at −20 °C until their use.

### 4.2. Chemicals and Reagents

For derivatization, a reagent prepared by BSA (*N*,O-bis(trimethylsilyl)acetamide), TMCS (trimethylchlorosilane), and TMSI (*N*-trimethylsilylimidazole) (3:2:3) was supplied by Supleco (Bellefonte, USA). A phosphate buffer was prepared with sodium dihydrogen phosphate and disodium hydrogen phosphate, both purchased from Panreac Quimica SLU (Barcelona, Spain). HPLC-grade solvents such as methanol, acetonitrile, ethyl acetate, and hexane, as well as sodium chloride (analytical grade), were acquired from Merck KGaA (Darmstadt, Germany). Sodium hydroxide was supplied by BDH Prolabo-VWR International (Barcelona, Spain). Creatinine standard and picric acid moistened with water (≥98%) were purchased from Sigma-Aldrich (St. Louis, USA).

### 4.3. Sampling

Urine samples were collected from esophageal cancer patients (case group; *n* = 17) with disease confirmation by pathological procedure and their healthy close relatives (control group; *n* = 10) in Golestan. The age ranges for the control and the case group were 20–46 (mean 33.5 years) and 50–92 (mean 69.1 years), respectively. Regarding the patients’ sex, the ratios of men and women were 9/1 and 10/7 in the control and the esophageal cancer group, respectively.

The first urine from early morning was collected in a polyethylene vessel, and samples were kept in a cool container (5 °C) during their transport to the laboratory. Urine samples were then prepared in 50 mL aliquots and kept frozen (−20 °C) until their analysis. The study was approved by the ethical committee at Golestan University of Medical Sciences. Participants filled in a web-based self-assisted diet record over one day and a questionnaire covering background information such as age, gender, marital status, history of squamous cell carcinoma (scc) in their family, other history of malignancy in the family, weight, height, and history of drug use, as well as a diet record of 24 h before urine samples focused on the amount and type of bread, dairy products, drink, rice, meat, vegetables, and fruits (Supplementary Materials). Written and approved informed consent was obtained according to the Helsinki Declaration (ethical principles for medical research involving human subjects).

### 4.4. Sample Preparation

#### 4.4.1. Extraction

Urine samples were defrosted and centrifuged (12,000 rpm, 5 min, 4 °C) before collecting 1 mL of the supernatant for mycotoxin extraction with a dispersive liquid–liquid microextraction (DLLME) procedure. Briefly, 0.3 g NaCl was added to each sample, and the samples were vigorously shaken. Then, 1 mL acetonitrile and 100 µL ethyl acetate were added, and the samples were vortexed for 1 mi, and centrifuged at 5000 rpm for 5 min at 4 °C. A supernatant layer was collected, and 50 µL of the extract was completely dried under nitrogen flow.

#### 4.4.2. Derivatization

Derivatization was carried out by adding 50 µL of BSA + TMCS + TMSI (3:2:3) to the dry extract, which was kept at room temperature for 30 min. Derivatized samples were diluted up to 200 µL with hexane and then thoroughly vortexed for 30 s. Afterward, 1 mL of phosphate buffer (60 mM, pH 7) was added, and the upper layer, containing the hexane phase, was transferred to a vial for the GC–MS/MS analysis.

### 4.5. GC–MS/MS Analysis

Simultaneous determination of all mycotoxins was performed by GC–MS/MS using a chromatographic instrument, Agilent 7890A, connected to a triple quadrupole mass spectrometer with inert electron-impact ion source (Agilent 7000A) and an Agilent 7693 autosampler (Agilent Technologies, Palo Alto, USA). Analyte separation was achieved on a 0.25 µm capillary column (HP-5MS 30 m × 0.25 mm). GC–MS/MS analysis was performed as described in a previously published manuscript [33]. Agilent Masshunter version B.04.00 software (Agilent Technologies, Palo Alto, USA) was used for data acquisition and processing. Two MS/MS transitions were acquired for each mycotoxin, using the most abundant for quantitation purpose, while the other transition served for confirmation process. Table 3 shows the retention time, quantitation (Q) and confirmation (q) transitions, collision energy, dwell time, and ion ratio for each mycotoxin.

### 4.6. Creatinine Analysis

Creatinine levels in urine samples were determined by spectrophotometry [44]. Briefly, alkaline picrate was formed by mixing picric acid (3.5 mM) and NaOH (1000 mM), and the solution was stored in darkness. Then, 1 mL of diluted urine (1/10, v/v, in ultrapure water) was mixed with 1 mL alkaline picrate, and optical density at 500 nm was measured in the spectrophotometer (Shimadzu mini 1240; Kyoto, Japan). Mycotoxin urinary concentrations were expressed as μg/g creatinine after normalization to creatinine level in the analyzed samples.

### 4.7. Method Validation

**Figure 2:**
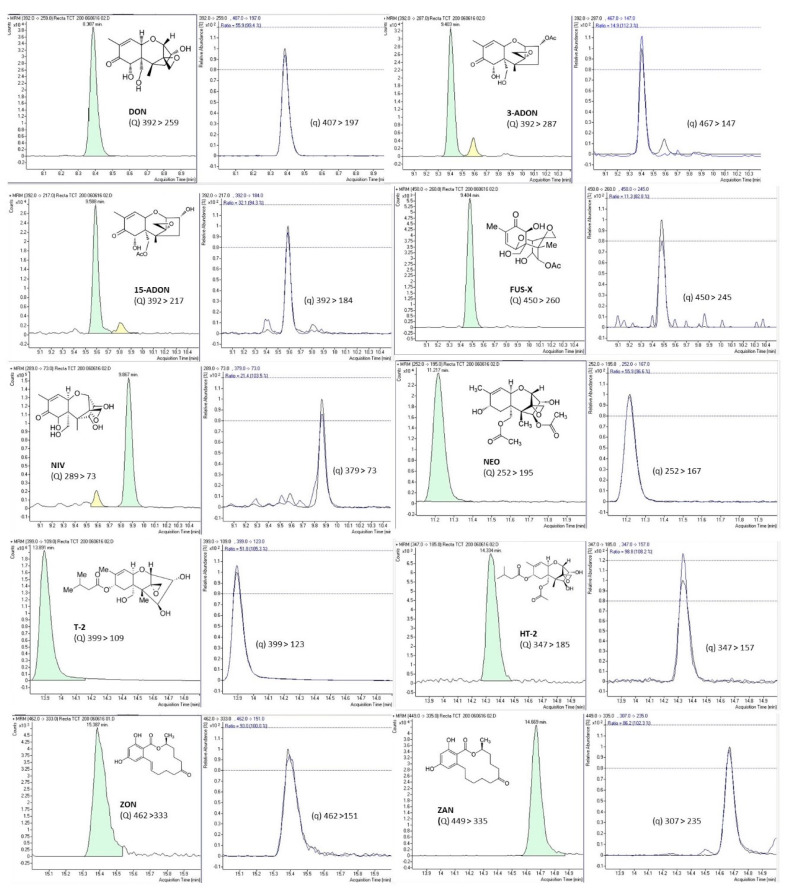
SRM chromatograms of the 10 studied mycotoxins from a urine sample fortified at 200 µg/L of each mycotoxin, as well as the chemical structure and the transitions used for quantitation (Q) and confirmation (q) transitions for each mycotoxin.

Validation of the analytical performance of the proposed method was implemented according to the European Union SANTE/12089/2016 guidance document on the identification of mycotoxins in food and feed to guarantee the quality of the analytical procedure [45]. Method performance for all tested mycotoxins was verified, including parameters such as accuracy (extraction recovery), repeatability or intraday precision, reproducibility or interday precision, linearity, matrix effect, limit of detection (LOD), and limit of quantitation (LOQ). Accuracy and precision were evaluated in triplicate by spiking blank samples (50, 100, and 200 µg/L) left to equilibrate overnight before the analysis. Accuracy was expressed as percentage of recovery of the spiked blank samples, while method precision, expressed as a percentage of relative standard deviation (RSD%), was verified by the triplicate analysis at the three studied concentrations (50, 100, and 200 µg/L). Precision included repeatability by performing the analysis on the same day (intraday precision) and reproducibility after three nonconsecutive days’ analysis (interday precision). To assess linearity and matrix effect, calibration curves of the studied analytes were prepared in pure solvent (external calibration) and in spiked blank urine samples (matrix-matched calibration). Both calibration curves were built by the peak areas and the corresponding standard analyte concentration at eight concentration levels ranging between LOQ and 100 times LOQ. Analytes’ relative ion intensities obtained in the standard solution and in the spiked samples were compared. Matrix effect was expressed as the percentage of signal suppression effect (SSE) or signal enhancement effect (SEE) by comparing matrix-matched calibration and external calibration curves. Analyte identification was based on the retention times of mycotoxins in both standards and urine samples, which were compared at a tolerance of ±0.5%. Table 4 shows the main validation parameters for the studied mycotoxins. Complete validation results are shown in a previous publication of the research group [33]. Figure 2 shows SRM chromatograms of the 10 studied mycotoxins from a urine sample fortified at 200 µg/L of each mycotoxin, as well as the chemical structure and the transitions used for quantitation and confirmation (Q and q) for each mycotoxin.

### 4.8. Statistical Analysis

Obtained data were statistically analyzed by the Student’s t-test of samples’ replicates (*n* = 3). No statistically significant differences for a confidence interval of 95% were found for all of the repeated measures.

## Figures and Tables

**Table 1:** Incidence; minimum, maximum, and mean mycotoxin concentrations (µg/L) with SD; and creatinine-corrected mean value (µg/g creat) of each detected mycotoxin in the control and esophageal cancer case groups.

Population Group	Parameters	DON	NEO	T-2	HT-2
Control (*n* = 10)	Positive samples	1	4	-	1
	Incidence (%)	10	40	-	10
	Min ± SD (µg/L)	-	10.57 ± 0.33	-	-
	Max ± SD (µg/L)	-	22.53 ± 2.39		-
	Mean ± SD (µg/L)	8.42 ± 1.21	14.15 ± 5.72	-	23.97 ± 9.97
	Corrected mean (µg/g creat)	5.90	10.45	-	16.81
Esophageal cancer (*n* = 17)	Positive samples	-	1	1	3
	Incidence (%)	-	5.8	5.8	17.6
	Min ± SD (µg/L)	-	-	-	18.91 ± 3.05
	Max ± SD (µg/L)	-	-	-	50.38 ± 22.54
	Mean ± SD (µg/L)	-	12.90 ± 3.87	50.09 ± 7.51	36.52 ± 16.07
	Corrected mean (µg/g creat)	-	9.09	44.70	29.09

**Table 2:** Co-occurrence of mycotoxins in the analyzed urine samples.

Mycotoxin(s)	Incidence (%)	Sample Group	Ʃ Mycotoxin Concentrations (µg/L)	Ʃ Mycotoxin Concentrations (µg/g Creatinine)
Binary Combination				
NEO/HT-2	1/27 (3.7)	Case	63.27	44.58
T2/HT-2	1/27 (3.7)	Case	90.38	79.13
Ternary Combination				
DON/NEO/HT2	1/27 (3.7)	Control	42.82	30.04

**Table 3:** Optimized parameters for gas chromatography–tandem mass spectrometry (GC–MS/MS) analysis of the selected mycotoxins.

Mycotoxin	RT (min)	Quantitation Transition (CE, eV)	Quantitation Transition Dt (ms)	Confirmation Transition (Collision Energy, eV)	Confirmation Transition Dt (ms)	Ion Ratio (%)
DON	8.39	392 > 259 (10)	25	407 > 197 (10)	25	41.6
3-ADON	9.40	392 > 287 (5)	35	467 > 147 (10)	25	47.5
15-ADON	9.58	392 > 217 (20)	35	392 > 184 (20)	20	35.5
FUS-X	9.484	450 > 260 (10)	35	450 > 245 (20)	35	11.9
NIV	9.867	289 > 73 (15)	35	379 > 73 (15)	35	29.6
NEO	11.22	252 > 195 (10)	25	252 > 167 (15)	35	40.6
T-2	13.891	399 > 109 (10)	25	399 > 123 (15)	35	81.9
HT-2	14.334	347 > 185 (10)	25	347 > 157 (10)	25	86.7
ZAN	14.669	449 > 335 (15)	25	307 > 235 (10)	25	59.9
ZON	15.387	462 >333 (20)	25	462 > 151 (20)	25	99.7

RT: retention time; CE: collision energy; Dt: dwell time.

**Table 4:** Main validation parameters for the studied mycotoxins.

Mycotoxin	Linearity (r^2^)	LOD (µg/L)	LOQ (µg/L)	Matrix Effect (% SSE)	Recovery Range (%)	Intraday Precision Range (%RSD)	Interday Precision Range (%RSD)
DON	0.996	0.12	0.25	23	88−97	2−4	8−10
3-ADON	0.992	0.25	0.5	27	84−102	1−9	4−11
15-ADON	0.991	0.25	0.5	28	77−91	2−10	3−12
FUS-X	0.992	2	4	12	83−95	3−6	6−13
NIV	0.996	0.5	1	6	82−95	3−7	4−7
NEO	0.999	0.25	0.5	36	93−109	5−7	3−11
T-2	0.998	0.5	1	8	89−104	4−5	8−10
HT-2	0.999	1	2	28	92−105	1−6	6−9
ZAN	0.993	4	8	36	72−80	2−5	5−12
ZON	0.991	3	6	23	79−96	6−7	8−11

## Data Availability

Not applicable.

## Supplementary Materials

The following are available online at https://www.mdpi.com/article/10.3390/toxins13040243/s1.
