# Supplementary material for: Biomonitoring of Multiple Mycotoxins in Urine by GC–MS/MS: A Pilot Study on Patients with Esophageal Cancer in Golestan Province, Northeastern Iran
Source: Toxins (Basel). 2021 Mar 29;13(4):243. doi: 10.3390/toxins13040243 (PMC8065391; doi:10.3390/toxins13040243)
Supplement: Supplementary file 1 [file toxins-13-00243-s001.pdf]

toxins

MDPI

Supplementary Materials: Biomonitoring of Multiple Mycotoxins in Urine by GC–MS/MS: A Pilot Study on Patients with Esophageal Cancer in Golestan Province, Northeastern Iran

Farhad Niknejad, Laura Escrivá, Khoda Berdi Adel Rad, Masoud Khoshnia, Francisco J. Barba and Houda Berrada

Questionnaire Biomonitoring of multiple mycotoxins in urine: Golestan University of Medical Sciences

1. Patients ID
2. Age
3. Gender: ☐Male ☐Female
4. Marital status:
5. Address:
6. History of SCC in family: ☐Yes ☐No
7. Other malignancy history in family: ☐Yes ☐No
8. Weight
9. Height
10. History of Drug use
11. Diet 24 h before urine samples

Bread	Amount	Types
Dairy Products	Amount	Types
Drink	Amount	Types
Rice	Amount	Types
Meat	Amount	Types
Vegetables	Amount	Types
Fruits	Amount	Types
